# Role of Promising Secondary Metabolites to Confer Resistance Against Environmental Stresses in Crop Plants: Current Scenario and Future Perspectives

**DOI:** 10.3389/fpls.2022.881032

**Published:** 2022-05-09

**Authors:** Delai Chen, Bismillah Mubeen, Ammarah Hasnain, Muhammad Rizwan, Muhammad Adrees, Syed Atif Hasan Naqvi, Shehzad Iqbal, Muhammad Kamran, Ahmed M. El-Sabrout, Hosam O. Elansary, Eman A. Mahmoud, Abdullah Alaklabi, Manda Sathish, Ghulam Muhae Ud Din

**Affiliations:** ^1^College of Life Science and Technology, Longdong University, Qingyang, China; ^2^Gansu Key Laboratory of Protection and Utilization for Biological Resources and Ecological Restoration, Qingyang, China; ^3^Institute of Molecular Biology and Biotechnology, The University of Lahore, Lahore, Pakistan; ^4^Department of Environmental Sciences and Engineering, Government College University Faisalabad, Faisalabad, Pakistan; ^5^Department of Plant Pathology, Bahauddin Zakariya University, Multan, Pakistan; ^6^Faculty of Agriculture Sciences, Universidad de Talca, Talca, Chile; ^7^School of Agriculture, Food and Wine, The University of Adelaide, Adelaide, SA, Australia; ^8^Department of Applied Entomology and Zoology, Faculty of Agriculture (EL-Shatby), Alexandria University, Alexandria, Egypt; ^9^Plant Production Department, College of Food and Agricultural Sciences, King Saud University, Riyadh, Saudi Arabia; ^10^Department of Food Industries, Faculty of Agriculture, Damietta University, Damietta, Egypt; ^11^Department of Biology, Faculty of Science, University of Bisha, Bisha, Saudi Arabia; ^12^Centro de Investigación de Estudios Avanzados del Maule (CIEAM), Vicerrectoría de Investigación y Postgrado, Universidad Católica del Maule, Talca, Chile; ^13^State Key Laboratory for Biology of Plant Disease and Insect Pests, Institute of Plant Protection, Chinese Academy of Agricultural Sciences (CAAS), Beijing, China

**Keywords:** PR proteins, polyamines, compatible solutes, antioxidants, stresses

## Abstract

Plants often face incompatible growing environments like drought, salinity, cold, frost, and elevated temperatures that affect plant growth and development leading to low yield and, in worse circumstances, plant death. The arsenal of versatile compounds for plant consumption and structure is called metabolites, which allows them to develop strategies to stop enemies, fight pathogens, replace their competitors and go beyond environmental restraints. These elements are formed under particular abiotic stresses like flooding, heat, drought, cold, etc., and biotic stress such as a pathogenic attack, thus associated with survival strategy of plants. Stress responses of plants are vigorous and include multifaceted crosstalk between different levels of regulation, including regulation of metabolism and expression of genes for morphological and physiological adaptation. To date, many of these compounds and their biosynthetic pathways have been found in the plant kingdom. Metabolites like amino acids, phenolics, hormones, polyamines, compatible solutes, antioxidants, pathogen related proteins (PR proteins), etc. are crucial for growth, stress tolerance, and plant defense. This review focuses on promising metabolites involved in stress tolerance under severe conditions and events signaling the mediation of stress-induced metabolic changes are presented.

## Introduction

**FIGURE 1:**
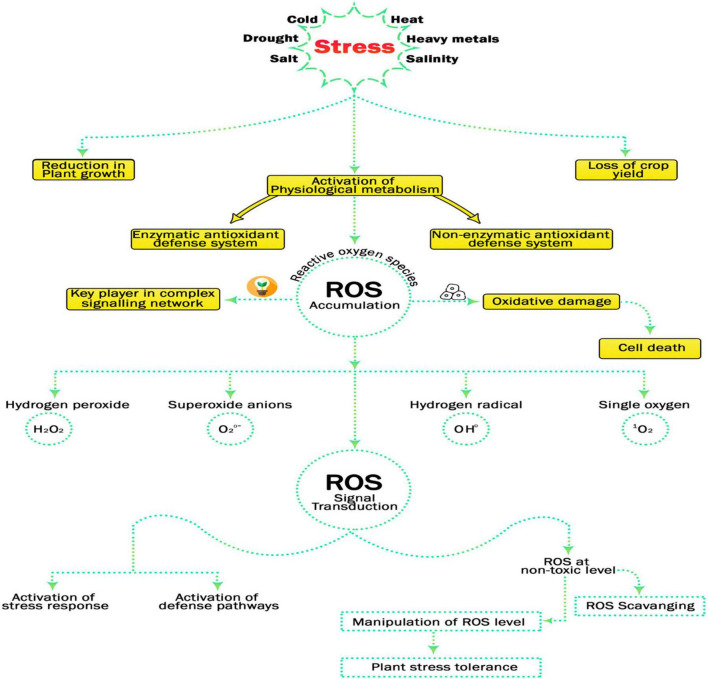
Importance of Reactive oxygen species in signal transduction and activation of defense pathways if at non-toxic level and its manipulation confers plant stress tolerance.

Plant activities are affected by multiple environmental factors. Some of them may act as stressors depending on their duration and intensity. Stress can be described as any adverse environmental condition affecting growth of the plant and crop quality and the final rate of return. Drought, frost, low and high temperatures, high humidity, acidity and salinity of the soil, and pollution by pesticides (Hasanuzzaman et al., 2013; Begum et al., 2019; Kamran et al., 2019a) have negative influence on plant growth and development which, in turn, inhibits metabolism, change their mood, and use internal energy to overcome the effects of stress. Forbearance or vulnerability to the stresses is extremely complicated affair. It can affect many stages of plant development, and many stresses disturb plants simultaneously (Iqbal and Ansari, 2020). Environmental stress imposed on plants can be categorized as abiotic stress and biotic stress, where the biological stress includes pathogen attack that may be caused by bacteria, fungi, nematodes, oomycetes, and herbivores (Poveda, 2021)while, abiotic stress includes salinity, drought, floods, extreme temperatures, heavy metals and radiation, etc. (Kamran et al., 2019b,^2021^; Ali et al., 2021; Parveen et al., 2021; Riaz et al., 2022). The diseases caused by a variety of biotic and abiotic stresses have led to a dramatic drop in global yield of crops (Etesami and Jeong, 2018). Drought and salinity disturb more than 10% of cultivated land, which results in a decrease in average world agricultural profits of more than 50% (Mahajan and Tuteja, 2005; Mustafa et al., 2019). This situation will worsen with the intensification of land-based desertification, soil and water salinization, water shortages and environmental pollution. When plants grow in stressful conditions, ROS are often produced as metabolic byproducts (Medina et al., 2021). When electrons from the electron transport chain in mitochondria and chloroplasts leak and react with the O_2_ molecule without other electron acceptors, ROS such as superoxide, hydrogen peroxide, hydroxyl and singlet oxygen are generated (Phua et al., 2021). Accumulation of reactive oxygen species (ROS) causes oxidative damage in plants (nucleic acids, proteins, and lipids) and causes degradation of chlorophyll pigments (El-Beltagi et al., 2020). Therefore, the generation of ROS files must remain within the limits of factory compatibility. ROS reaches to toxic levels under all forms of abiotic stress, can cause explosions of antioxidants in plant cells. A complex network of defense and repair mechanisms counter these oxidation reactions (Masamba and Kappo, 2021), however, any difference between ROS production and safe detoxification indicates a metabolic state called oxidative stress which causes oxidative damage to the plant (Figure 1).

There is a proverb; “Necessity is the mother of invention” it seems true in case of plants as they are sessile organisms, so they use a bunch of chemicals to stop enemies, resist pathogens, replace competitors, exceed environmental limits, and overcome oxidative stress (Ahuja et al., 2011). Plants combat harsh environmental circumstances in a complex and integrated way, as a function of time. Stress tolerance in plants concerns whole plants, tissues, cells, physiological and molecular levels. The unique combination of intrinsic changes determines the ability of plants to sustain themselves in adverse environmental situations (Farooq et al., 2009; Meena et al., 2017). This includes a variety of physiological and biochemical modifications in plants together with leaf wilt, leaf abscission, leaf area reduction, stimuli of root growth, relative moisture content variations, electrolyte outflow, the creation of reactive oxygen species and the accretion of free radicals, which interrupt homeostasis of cells, resulting in lipid peroxidation, damage of the membrane and enzymes inactivation, thus affecting the cell viability (Sebastiani et al., 2016; Giordano et al., 2020).

The advanced plants have wide-ranging mechanisms to protect in contrast to a variety of fears or dangers, comprising physical, biological and chemical and stresses. They have developed various defense mechanisms at different levels to cope with adverse environments. Prefabricated defense systems include the production of antibacterial molecules such as keratin, wax, and deposition of firm lignin on cell wall and plant antibacterial agents. They are often considered the first line of defense against a new attack of pathogens (Bieniasz, 2004; Cseke et al., 2016). For example, in case of water stresses sequence of biochemical and physiological feedbacks in plants start. These feedbacks consist of closure of stomata, check of photosynthesis and cell growth, and initiation of respiration (Lovisolo et al., 2010). Plants also react to and adjust to sliding defects at the cellular and molecular stages, including permeabilization and accumulation of proteins that specifically contribute to stress tolerance. Different genes with different tasks are stimulated or inhibited by these stress conditions (Slade and Radman, 2011). There are two key premeditated responses to stress effects: avoiding stress effects due to physiologically inactive phases and creating stress tolerance or coping skills with the help of their metabolites (Aertsen and Michiels, 2004). In this review, the aim is to highlight on environmental stress including the basics of stress resistance and the potential of plants with the help of their metabolites to improve their functioning by making them stress resistant.

## Metabolites Involved in Stress Tolerance

**FIGURE 2:**
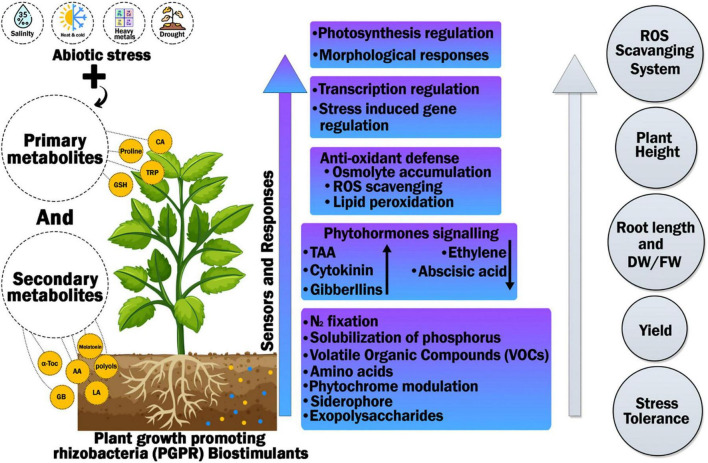
Response of primary and secondary metabolites in plants to various abiotic stresses to help plant in activation defense system and signal transduction.

Plant metabolites have many functions. They act as signal or regulators, well-suited solutes, antioxidants, or defenses against pathogens. Plant metabolites are responsible for stress tolerance in plants. The accretion of metabolites frequently arises in plants under stress from numerous elicitors or signaling molecules, as it is recognized that growth conditions like temperature, nutrient supply and lightning conditions influence the accumulation of different natural products (Ballhorn, 2011; Rini Vijayan and Raghu, 2020). Furthermore, more serious environmental impacts, like several stress conditions, will also influence on metabolic pathways liable for gathering plant’s secondary product (Shulaev et al., 2008; Chandran et al., 2020). Plants have two types of metabolites primary and secondary metabolites. Primary metabolites generally have the same biological function as all species, while the secondary metabolites are generally produced in plants to meet specific needs, as secondary metabolites are produced by modified synthetic pathways from primary metabolites or by sharing substrates of origin of primary metabolites (for example, carbohydrates, lipids, and amino acids) (Pott et al., 2019). Primary metabolites comprise of organic acids, acyl lipids, carbohydrates and phytosterols. Comparing to it, secondary metabolites come in action in special cases, and these are used as classification markers because of the limited distribution in taxonomic groups, nonetheless, these compounds are present in all plant tissues and have a metabolic activity for plant growth and development (Harwood, 2012). There is no clearly defined boundary between the two classes, and these cannot be separated according to their chemical structure, their precursor molecules and their sources of biosynthesis (Schwarzenbach et al., 2016), for example proline, an amino acid, is the main metabolite, while its C6 comparable molecule, pipecolic acid, is the alkaloid. Likewise, di-terpenes and tri-terpenoids contain primary and secondary metabolites (Edreva et al., 2008). Plants evolved to adapt to the environment, genetically coding useful synthesizes and various secondary metabolites (Janská et al., 2010; Rastegari et al., 2019; Figure 2).

It has also been shown that some of these compounds have an effect herbivores and appeal pollinators, allelochemicals and protective effects. Prevent toxicity, UV rays defending and signal transduction. Kossel named these chemicals as “secondary metabolites” in 1891 and described these organic compounds as essential to plant life (Ahmed et al., 2017). Secondary metabolites were considered biologically unimportant, so plant biologists paid slight consideration to them. However, since 1950s organic chemists have conducted extensive research on their structure and chemical properties (Mauseth, 2014). It is now clear that this belief is ambiguous and mistaken, and that secondary metabolites work and perform an important role in latent defense mechanisms, especially in chemical combat or competition between plants and pathogens (Croteau et al., 2000; Srivastava et al., 2021).

Overall, we can classify these metabolites into 3 major categories associated with stress tolerance in plants which include biochemicals, plant growth regulators and enzymes. The review will give insights in to the role of each of them in plant defense response under stress conditions.

## Proficiency of Biochemical Associated With Stress Tolerance

When plants are susceptible to stressors, their cells defend themselves from elevated concentrations of intracellular salts by mounting up small organic metabolites (collectively called compatible solutes). Compatible solutes have low molecular weight water, soluble in water and non-toxic even at higher concentrations. Compounds these are also called “osmolytes” (Chen and Murata, 2002; Bajguz and Hayat, 2009). Accumulation of these solutes is the common practice used to fight against environmental stress factors (Ma et al., 2010), where betaines, polyols, amino acids such as proline, sugars like mannitol, trehalose and sorbitol and polyamines are most common compatible solutes. Their accumulation is advantageous in case of deficiency of water or salt stress because they offer a tolerance to the stress of the cells without interfering with the cellular mechanisms. Tolerant or sensitive species exhibit differential stress tolerance, depending on the level of accumulation of these compounds during abiotic stress (Obata and Fernie, 2012).

### Glycine Betaine

Glycine betaine (GB) is the most important compatible solute and there are many reports of different roles of glycine betaine in higher plants like barley, corn, spinach, and sugar beet (Chen and Murata, 2008). Glycine betaine is effective in stabilizing the quaternary structure of enzymes and complex proteins and in protecting various components of photosynthetic machinery, such as ribulose-1,5-bisphosphate carboxylase/oxygenase (Rubisco) and oxygen release in photosystem II (PSII) and maintains a highly ordered membrane state at non-physiological and elevated salt concentrations (Aimon et al., 2011; Suo et al., 2017). The exogenous administration of glycine betaine and the introduction of the glycine betaine biosynthesis pathway in natural non-accumulation by transgenes increase the tolerance of these plants to various types of abiotic stress (Iqbal et al., 2011). This increased tolerance to abiotic stress is a useful system for studying the mechanisms by which GB protects plants from abiotic stress (Chen and Murata, 2011).

#### Synthesis of Glycine Betaine

Glycine betaine (GB) is synthesized by two different routes from two dissimilar substrates, like choline and glycine. The biosynthesis of glycine betaine from choline has been studied in animals, plants, and microorganisms. This pathway includes one or two enzymes, depending on the mode of oxidation of choline. Glycine betaine is the result of a two-step oxidation of choline by betaine aldehyde which is a toxic intermediate (Ashraf and Foolad, 2007). In higher plants first step is catalyzed by manoxygenase (CMO). The second oxidation step is catalyzed by NAD^+^ dependent betaine dehydrogenase (BADH) (Annunziata et al., 2019). The biosynthesis of GB is induced by stress, and the concentration of GB *in vivo* varies among plant species and is between 40 and 400 mol g-1 (DW) in natural accumulation under stress conditions (Wani et al., 2013).

#### Mechanism and Protective Role of Glycine Betaine

The accumulation of glycine betaine (GB) in plants is important to prevent oxidative stress induced by abiotic factors. In addition to osmotic protection, GB also enhances the antioxidant defense mechanism against stress damage (Hoque et al., 2008), asthe anticipated GB mediated abiotic stress tolerance mechanisms consist of stabilization of the natural structure of proteins and enzymes, osmotic regulation, integrity of membrane, fortification of photosynthesis, and detoxification of ROS formed all through stress. It could be assumed that GB protection involved in transcription and translation mechanisms may be facilitated by the initiation of specific gene expression under stress conditions, and that the products of these conditions are involved in the development of stress tolerance. GB has shown that *in vitro* melting temperature of double stranded DNA reduces (Rajendrakumar et al., 1997). This will allow GB to regulate gene expression by activating replication and thus transcription in a highly saline environment.

In addition, Bourot et al. (2000) have revealed that glycine betaine acts as a chaperone protein *in vivo*; hence, this suggests that GB can stabilize mechanisms of transcription and translation to efficiently express genes under stress environments. *In vitro* studies indicate that GB itself has no antioxidant activity (Smirnoff and Cumbes, 1989), in fact, acts indirectly by inducing the synthesis or activation of the ROS defense system. This has been demonstrated in exogenous accumulated GB and transgenic plants which store glycine betaine. Hoque et al. (2007) studied the effect of GB exogenous administration on antioxidant levels and enzymatic activity in the glutathione (GSH) ascorbate cycle (ASC-GSH cycle) in cultured tobacco suspension culture cells exposed to salt stress. They confirmed that salt stress significantly reduced ASC and GSH levels, as well as the activities of the ASC-GSH cycle enzymes, like ascorbate peroxidase (APX), monodehydroascorbate reductase (MDHAR), dehydroascorbate reductase (DHAR) and glutathione reductase (GR). In addition to MDHAR, the exogenous administration of GB increases the activity of all these enzymes. However, under normal conditions, GB has no direct positive effect on enzyme activity in the ASC-GSH cycle, but only in the presence of salt stress. Thus, enzyme activity in the ASC-GSH cycle, which enhances GB, protects tobacco cells from salt-induced oxidative stress.

Yang et al. (2007) discovered a link between the ability of transgenic tobacco plants to accumulate glycine betaine under heat stress and ROS production. ROS production increased dramatically, but that the yield of transgenic plants was much lower than that of the wild type, indicating that *in vivo* GB accumulation can reduce ROS production by case of heat stress. The improvement in the photosystem II (PSII) repair process induced by GB accumulation can be explained by the increased activity of a series of antioxidant enzymes and by the lower production of ROS in transgenic plants during heat stress.

### Amino Acids

Amino acid accumulation has been observed in many studies of plants exposed to abiotic stress (Lugan et al., 2010). The accumulation of amino acidsmay result due toincrease in degradation of stress-induced proteins. Although the accretion of amino acids under stress conditions indicate cellular damage in some species (Slama et al., 2015), the increase in specific amino acid levels has a beneficial effect during adaptation to stress. Among different amino acids, proline is predominantly abundant in plant species; however, in various taxonomic groups it is aggregated in response to biotic and abiotic and biotic stress (Szabados, 2010).

#### Proline

Proline is a group of tiny compounds known as osmolytes or osmoprotectants, therefore, as an osmolyte, proline is an important molecule used by various organisms to combat stress (Judy and Kishore, 2016). Proline levels differ from one species to the next, and proline concentration might be 100 times higher in dry conditions than in wet conditions. Proline is thought to protect membranes and proteins from high concentrations of inorganic ions and harsh temperatures, as well as to stabilize cellular structures, detoxify free radicals and store carbon (C), nitrogen (N2), and energy (Hare and Cress, 1997). Studies have also shown that proline can also modify the activity of antioxidant enzymes (Verbruggen and Hermans, 2008; de Campos et al., 2011). Proline (Pro) is thought to be involved in the non-enzymatic antioxidant defense of plants. The accumulation of Pro in stressed plants is up to 100 times higher than normal and has a history of more than 40 years. In this case, Pro can reach a cytosol concentration of 120 to 230 mM (Sytar et al., 2013).

#### Proline Synthesis

In plants subjected to osmotic stress, proline is mainly synthesized from glutamic acid by D1-pyrroline-5-carboxylate (P5C) by two consecutive reductions catalyzed by P5C synthase (P5CS), which is a rate-limiting enzyme of the biosynthetic pathway in complex plants and P5C reductase (P5CR) (Verbruggen and Hermans, 2008).

#### Mechanism and Defensive Role of Proline

Proline appears to act as a metabolic signal, regulating the pool of metabolites and affecting plant growth and development (Verbruggen and Hermans, 2008). Alternatively, proline may be conferred by induction of a stress protection protein (Chinnusamy et al., 2005). In fact, proline synthesis may be one of the first metabolic reactions triggered by signal transduction pathways, linking many perceptions of environmental stress to clarification of physiological responses at the cellular level (Song et al., 2005). Therefore, clarifying the change in proline content is a signal component, and the way it regulates gene expression, and the metabolic process will undoubtedly be a challenge and require further exploration. Proline accumulation in the drought-prone leaves is associated not only with increased expression of the P5CS gene, but also with decreased expression of the proline dehydrogenase (PDH) gene encoding a proline-degrading enzyme (Kishor et al., 2005; Yang et al., 2010). It has been determined that the metabolism of proline leads to improved production of mitochondrial reactive oxygen species (ROS) by means of the electron transport chain (ETC) and that proline metabolism affects survival and cell death of diverse species. In plants, the protective effect of proline during stress is particularly evident. Glutamate seems to be a major precursor of stress-induced proline accretion in plants, as the ornithine pathway primarily promotes the nitrogen cycle from arginine to glutamate. The proline synthesis limiting enzyme is P5CS and its increased expression is associated with proline accumulation in Arabidopsis (Sengupta et al., 2016).

**FIGURE 3:**
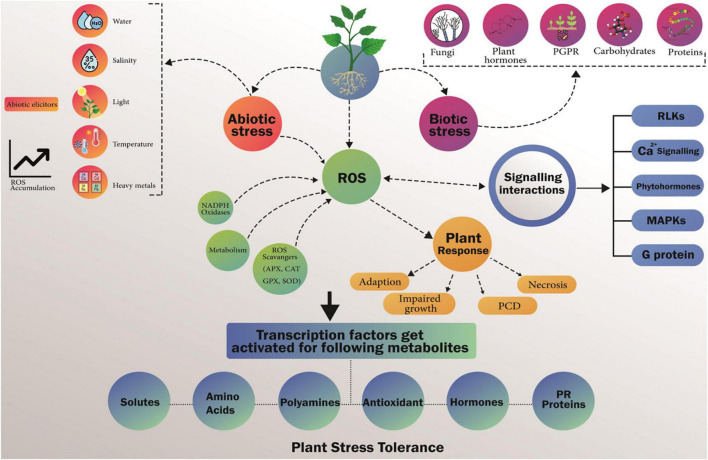
Hypothetical outline of biotic and abiotic stress factors and metabolites produced in response to encounter these factors and create stress tolerance. Metabolites production is up regulated in reaction to a number of biotic and abiotic stresses. This shows significance of metabolites in synchronize plant responses to environmental stresses. Environmental stresses stimulate sensors/receptors due to which reactive oxygen species (ROS) are produced. Variety of metabolites is produced to overcome stress produced by ROS which ultimately initiate cascades of regulatory mechanism resulting which transcription factors get activated.

The signaling mechanism of proline biosynthesis induced by environmental stress in plants comprises several molecules, such as abscisic acid (ABA), calcium and phospholipase C (Ahuja et al., 2010). Recently, Sharma et al. (2005) reported that the protection of ABA-induced growth in water-deficient plants requires proline metabolism. Evidence for ROS regulation of proline biosynthesis has also been found. Fabro et al. (2004) reported that in Arabidopsis, HR triggered by incompatible interactions between phytopathogens led to proline accumulation by up regulating P5CS2 rather than P5CS1 and ROS-dependent pathways in salicylic acid. Later, Abass and Mohamed (2011) also reported that hydrogen peroxide (H_2_O_2_) causes proline accumulation or promotes ABA-induced proline accumulation. Recently, Yang et al. (2012) suggested that H_2_O_2_ may induce proline accumulation by up regulating P5CS protein and down-regulating proline dehydrogenase 1 (PRODH) activity in coleoptile and radicle of maize seedlings (Figure 3).

In fact, it has been found that some plant tissues maintain the oxidation of proline under stress. The molecular mechanism by which proline protects cells during stress is not fully understood, however, it seems that proline implies its chemical properties and effects on redox systems, such as glutathione libraries (Sharma and Dietz, 2006). The role of proline in stress adaptation is often explained by its ability to penetrate and its ability to balance water stress. However, adverse environmental conditions often disrupt intracellular redox homeostasis and therefore require mechanisms to balance oxidative stress. Therefore, it is also proposed that the mechanism of proline protection involves the stabilization of antioxidant proteins and enzymes, the direct clearance of ROS and the balance of intracellular redox homeostasis (e.g., the NADP^+^/NADPH ratio) then metabolism of proline promotes cellular signaling. As a result, the potential mechanism by which proline provides protection against stress takes place (Liang et al., 2013).

Numerous studies have examined the role of proline in the osmotic protection of plants and its application in improving tolerance to water stress (Poustini et al., 2007), by using genetic transformation techniques, by genetic identification in proline accumulation under conditions of water deficiency or exogenous proline (Ashraf and Foolad, 2007). Although several studies have suggested that proline accumulation is important for abiotic stress tolerance, these findings are not conclusive. It may help crops develop resistance that have been subjected to abiotic stress (Kishor et al., 2005).

Although the clear relationship between proline accumulation and stress adaptation has been problematic, it is widely accepted that an increase in proline content after stress is beneficial for plants (Verbruggen and Hermans, 2008). Very high levels of cellular proline (up to 80% of the amino acid pool under stress and 5% under normal conditions) were recorded due to increased synthesis and reduced degradation under various stress conditions in many plant species (Szabados and Savoure, 2010).

**FIGURE 4:**
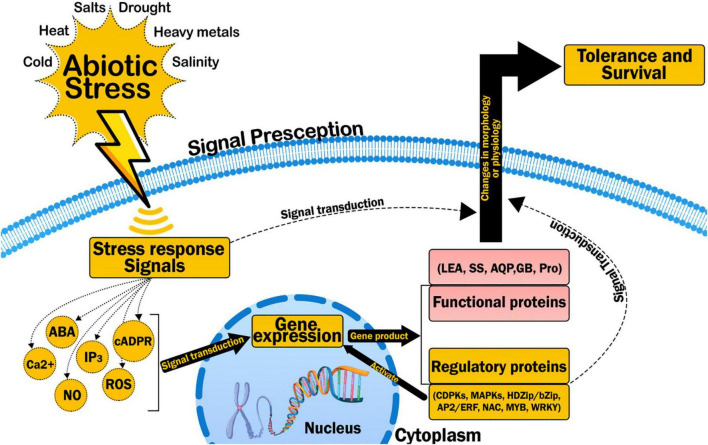
Activation of stress response genes and finally stress response is engineered by expression of these genes.

As a multifunctional amino acid, proline appears to have different effects under stress conditions, such as the stabilization of proteins, membranes and sub cellular structures, and the protection of cellular functions by the trapping of reactive oxygen species (ROS). Synchronization of proline biosynthesis and degradation of cytoplasm, chloroplasts and mitochondria increases the complexity of the diversification of proline metabolism. The rate of improvement of proline biosynthesis in chloroplasts can help stabilize the redox balance and maintain cellular homeostasis by eliminating the potential for excessive reduction when the electron transport chain is saturated under adverse conditions (Taiz and Zeiger, 2010). The catabolism of proline in mitochondria is associated with oxidative respiration and energy is applied after stress to restore growth. Although pretreatment with proline can improve the phytotoxicity of heavy metals by reducing the concentration of ROS (Viehweger, 2014), it can also be used as a protein-compatible hydrotrope (Verslues and Sharma, 2010) to reduce cytoplasmic acid. Poison and maintain an appropriate NADP ^+^/NADPH ratio compatible with cellular metabolism (Filippou et al., 2014). Proline offers tolerance to various abiotic stresses by increasing its endogenous levels and its intermediate enzymes in the plant. Exogenous proline administration has been reported to increase endogenous levels of proline in bean (*Phaseolus vulgaris* L.) (Aggarwal et al., 2011). Proline regulates the expression of antioxidant enzymes on plant exposure to salt stress. Among various genes, Δ1-pyrroline-5-carboxylic synthase genes are responsible for the up-regulation of stress-induced proline under the effect of salt stress (Qamar et al., 2015; Figure 4).

### Polyamines

Polyamines are recognized as a group of naturally occurring compounds which have aliphatic nitrogen structure, present in almost all organisms and playing a significant role in many physiological processes, such as cell growth and development and the response to environmental stress. Putrescine (Put), spermidine (Spd) and spermine (Spm) are the most common polyamines in higher plants and may exist in free, soluble, conjugated, and insoluble combinations. In plants, larger accumulation of polyamines (Put, Spm and Spd) in the course of abiotic stress is well acknowledged and is associated with improved tolerance to abiotic stress (Bitrián et al., 2012). The metabolic pathway of polyamines is one of the pathways found in normal-stage plants (organogenesis, embryogenesis, flower and fruit development and senescence), whose main function is to stabilize macromolecular structures and regulate various environmental stimuli of biological properties such as biotic and abiotic factors (Kusano et al., 2007). Polyamines regulate the response of plants to broader expected responses to abiotic stress, such as drought, salinity, heavy metal toxicity, oxidative stress, cooling, elevated temperatures, osmotic stress, leeches, and tolerance to flood, as evidenced by the exogenous application. Polyamines or transgenic plants developed to express genes involved in the biosynthesis of polyamines. Since polyamines are regulated by various abiotic environmental stimuli, their cellular functions are: mineral nutrient deficiencies, metal toxicity (Alcázar et al., 2006; Choudhary et al., 2012), salinity, high and low temperatures (Hummel et al., 2004), drought (Alcázar et al., 2010), hypoxia (Moschou et al., 2008), infiltration (Osmotic) and oxidative factors (Gill and Tuteja, 2010). In addition to regulating the titer in response to external stimuli, polyamines also modify ion channels (Takahashi and Kakehi, 2010), stimulate the synthesis of specific proteins, stimulate the assembly of 30S ribosomal subunits and stimulate the formation of Ile-tRNAs (Igarashi and Kashiwagi, 2000).

Polyamines are the cationic compounds, having two or more amine groups, which are low molecular weight organic molecules present in most organisms with multiple functions owing to the diversity of the number and position of the amino groups. Due to this cationic nature of the polyamines, they readily bind to the DNA, RNA and proteins are produced by electrostatic connection which results in either stabilization and equilibrium or destabilization (Kusano et al., 2008). There is a covalent connection of polyamines to numerous enzymes or proteins (post-translational modifications) which takes part in physiological processes was catalised by trans-glutaminase under normal or stressful conditions (Folk et al., 1980).

**FIGURE 5:**
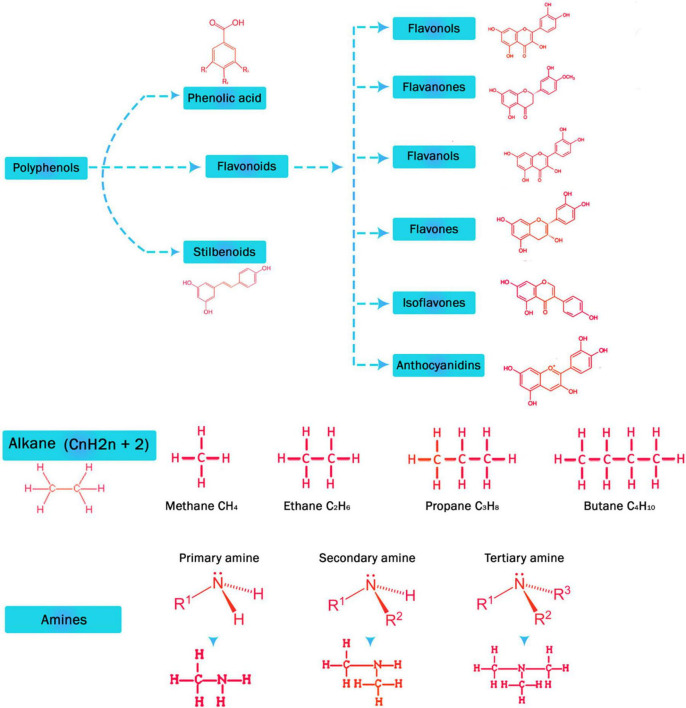
Role of polyamines in various physiological processes occurring in plants regulate defense pathways and their cascade of tolerance to heavy metals.

Moreover, it has been reported that the regulated titer of polyamines binds to epibrassinolide (active form of brassinosteroid) and modulates the pathways of abscisic acid (ABA) and indole-3-acetic acid (IAA), thus reinforcing Tolerance to toxicity (Choudhary et al., 2012). In addition to the fact that heavy metals themselves are toxic to plants, they also stimulate oxidative stress because heavy metals are essentially ionic. The polyamines associated with brassinosteroids, in addition to regulating the pathways of ABA and IAA and their cascade of tolerance to heavy metals; also regulate antioxidants such as glutathione, ascorbic acid, valine, glycine- betaine, etc. Antioxidant levels and enzymes such as glutathione reductase, superoxide dismutase, catalase, peroxidase, etc., impart stress tolerance (Chaudhary, 2012; Figure 5).

Improved polyamine levels by exogenous feeding (Gill and Tuteja, 2010) or by heterologous expression of polyamine biosynthesis genes in transgenic plants have been shown to improve abiotic stress tolerance (Liu et al., 2007). Endogenous polyamine titers (in particular Put, Spd and Spm) are regulated not only by the regulatory gene expression pattern of polyamine biosynthesis genes, but also by the regulated expression of genes involved in catabolism of polyamines enzyme responsible for catalyzing oxidative deamination of polyamine having its peculiar function. In other respects, one may even speak of the biosynthetic pathway of H_2_O_2_, since it has been reported that in plants, H_2_O_2_ is differentiated by the oxidation of polyamines, which shows lignification during development of cell wall and ontogeny and also aids fight against biotic and abiotic stresses (Wimalasekera et al., 2011). The induction of most genes associated with polyamines biosynthesis during either abiotic stress proposes a seemingly reasonable functional relationship between PA metabolism and abiotic stressors (Shi et al., 2013). In addition, ABA induces polyamine biosynthesis genes, which is a known stress regulator, which acts upstream of the polyamine biosynthetic pathway (Alcázar et al., 2006); the abiotic stress tolerance of plant species is improved, reinforcing the close relationship between PA and stress (Sagor et al., 2013).

Although a clear picture of the role of polyamines in abiotic stress is emerging, their critical role in tolerance to abiotic stress remains complex, as the polyamine’s substrate is also affected by other key metabolites like ethylene. The deactivation of the gene for ethylene biosynthesis, ACC synthase and ACC oxidase, is accompanied by an increase in polyamines (Put and Spd), leading to an increase in the abiotic tolerance of tobacco plants (Naing et al., 2021). In addition, the regulation of arginase, a nitrogen metabolizing enzyme, leads to changes in polyamines (Put and Spm), thus affecting the improvement of abiotic stress tolerance (Shi et al., 2013). Likewise, modulation of PAO (an enzyme associated with catabolism of polyamines) leads to salt tolerance in tobacco (Moschou et al., 2012). Adding to the above, the combined network of ABA, NO and PA associated with abiotic stresses has also begun to present interaction groups associated with regulation of abiotic stress tolerance. By catabolism of PAs (Spd and Spm) under abiotic stress by copper amine oxidase (CuAO) and phyllobilin (PAO) (Wimalasekera et al., 2011), it was observed that ABA induces PA accumulation (Alcázar et al., 2010) and NO synthesis in different plant species, thus revealing the complex interaction of these three metabolites, namely ABA, No and polyamines. Furthermore, the cell balance of PA in cells is mainly determined by the regulation of their biosynthesis and catabolism. In plants, PA concentration is much higher than that of plant hormones but is considered a growth regulator because of its different roles in plant growth & development (Menéndez et al., 2013).

Zapata et al. (2004) studied the special effects of salinity ongrowth of the plant, ethylene production and polyamine content in spinach, lettuce, cucumber, pepper, cabbage, sugar beet and the tomato. They found that polyamine levels varied with salinity and, in most cases, decreased and increased spermidine and spermine levels. The ratio of spermidine, spermine and putrescine increased with the increase in salinity resulting in tolerance to salinity for all species. The results showed that the general response of different plant species to PA production was related to PA production, but not to ethylene production. Meanwhile, Liu et al. (2004) stated that PEG 6000 treatment expressively increased levels of free Spermidine and free Spermine in drought-resistant wheat leaves. They suggested that free spermidine, F, free spermine and PIS-linked Put favor osmotic stress tolerance in wheat seedlings.

### Antioxidants

As discussed before that reactive oxygen species (ROS), like superoxide radicals (O_2–_), hydrogen peroxide (H_2_O_2_) and hydroxyl radicals (∙OH) are formed during the common aerobic metabolism. ROS are manufactured in changed cellular components, for example, cell walls, plasma membranes, chloroplasts, mitochondria, and peroxisomes (Dat et al., 2000). Mainly, these oxygen species are extremely reactive, resulting in protein lipids, DNA damage and eventually cell death (Foyer and Noctor, 2005). The concentration of reactive oxygen species formation is determined by the interaction between ROS production and scavenging capacity of ROS. Abiotic stress causes excess production of reactive oxygen species. The excessive production of ROS leads to oxidative damage (Fridovich, 1986). To avoid oxidative damage, plants use non-enzymatic antioxidants like ascorbic acid (AsA) and glutathione (GSH), as well as enzymatic antioxidants such as superoxide dismutase, catalase, ascorbate peroxidase (APX) and glutathione reductase (GR) to remove ROS (Noctor and Foyer, 1998).

#### Glutathione

Antioxidants are a key factor in protecting plants against oxidative damage initiated by abiotic stress. Among other non-enzymatic antioxidants glutathione (GSH; c-glutamyl-cysteinyl-glycine) is a low molecular weight, water soluble thiol compound widely used in most plant tissues. In addition to its role in reducing the storage and transport of sulfur, GSH is directly or indirectly involved in the detoxification of ROS (Foyer and Noctor, 2005). Along with detoxification of ROS, GSH is also involved in the detoxification of methylglyoxal (MG) (Hasanuzzaman et al., 2017a,b). It works as a cofactor for different biochemical reactions, interacting with hormones, signaling molecules and its redox state elicits signal transduction (Foyer and Noctor, 2005). One more major function of GSH is the formation of phytochelatines (PCs), which bind heavy metals for harmless transport and repossession in vacuoles (Sharma and Dietz, 2006). As a result, it plays a vibrant role in the detoxification of toxic metals, metalloids and xenobiotics (Srivalli and Khanna-Chopra, 2008). GSH contains several biochemical properties that enable it to aid plant growth and development in a range of settings, including normal growing conditions and stress. Thus it regulates cell proliferation, apoptosis, fibrogenesis, growth, development, cell cycle, gene expression, protein activity, and immunological functions (Shao et al., 2008; Nahar et al., 2015). Glutathione S transferase is second phase enzyme in detoxification. To study the stress response of Glutathione S- transfrase (GST) at the level of transcription in plants, a complete study of the culture of the Arabidopsis cell suspension was conducted. The analysis indicates that early changes induced by stress in gene expression led to functional redundancy and precise GST involvement in protection against oxidative stress (Sappl et al., 2009).

In another study, the chloroplast antioxidant defense system showed improvement due to increased gene expression of DHAR (Dehydroascorbate reductase) and GST enzymes (Le Martret et al., 2011). Moreover, the molecular docking of SlGSTU5, a member of GST for tomato and one of the ligands, indicates that SlGST activity can enhance crop improvement and stress resistance by using a defense agent (Islam et al., 2017).

#### Tocopherols

It is also called vitamin E and it is liposoluble antioxidants that bind to membranes and are therefore not only worthy scavengers for oxides but also for lipid peroxides (Blokhina et al., 2003). Tocopherols consist of four isomers such as α, β, γ and δ. Among these α-tocopherol has the primary antioxidant aptitude. Tocopherols are produced by plants and confined in chloroplasts. Their shielding effects have been studied and confirmed under the influence of stress such as drought, salinity, or heavy metals (Gill and Tuteja, 2010).

Tocopherol accumulation differs significantly among plant species and sections. Tocopherols have several roles in plant development and physiological processes, many of which have an impact on yield. Tocopherols are essential for abiotic stress tolerance (e.g., salinity, drought, metal toxicity, ozone, UV radiation). According to several studies, stress-tolerant plants have higher levels of tocopherol, whereas sensitive plants have lower levels, resulting in oxidative damage. Tocopherols capacity to scavenge or quench lipid peroxides, oxygen radicals, or singlet oxygen, resulting in ROS detoxification, is the most significant role of tocopherols in alleviating abiotic stress-induced damage. Mutant and transgenic techniques that result in the loss or increase of function of tocopherol pathway genes likely to shed light on the compound’s role in plants (Waqas et al., 2019; Zandi and Schnug, 2022). ROS are produced in response to various abiotic stimuli including dryness, salt, low temperature, and heavy metals, which harm membrane-containing organelles. To deal with ROS, tocopherols (a lipid-soluble molecule) quenches and detoxifies these ROS radicles. Although the biosynthetic enzymes for tocopherols have been cloned and researched extensively, our understanding of the whole regulation of this process in plants under environmental conditions is still lacking (Yang et al., 2021).

Novel insights into the control and introgression of the tocopherol processes in plants were gained from research in biosynthetic mutants and metabolic engineering of tocopherol pathways. Attempts to increase total tocopherol levels by genetic modification, on the other hand, have mostly failed. The reason is that tocopherol levels grow several times more under abiotic stressors than in untreated Arabidopsis leaves. This suggests that the potential for high tocopherol levels significantly surpasses what has been genetically produced so far (Ali et al., 2022).

#### Ascorbic Acid

It is also called vitamin C. It is a small water-soluble and it is regenerative by nature thus it is highly effective antioxidant molecule (Noctor and Foyer, 1998). It can be used as a major substrate in the cyclic pathway for the enzymatic detoxification of many reactive oxygen species (ROS), like H_2_O_2_ and many other plants that are harmful to normal functions of plant’s metabolism. In addition, it acts directly on neutralizing superoxide radicals (O2∙-), singlet oxygen (O∙−) or hydroxyl radicals (OH∙−). This antioxidant activity of ascorbic acid is associated with oxidative stress and long life of the plant. In addition, it has recently been suggested that endogenous levels of ascorbic acid are important in the regulation of developmental senescence and the defense of plants against pests (Khan et al., 2011). Numerous studies have reported regulation of metabolism of antioxidant defense by ascorbic acid in many plants that were grown under stress factors. Such as canola (Bybordi, 2012), *Abelmoschus esculentus* (Raza et al., 2013) and *Hordeum vulgare* (Agami, 2014) under extreme saline condition as well as *Brassica napus* in drought condition (Shafiq et al., 2014) and wheat plant under lead toxicity (Alamri et al., 2018). Association of ascorbic acid has been reported in stimulation of various antioxidant enzymes. Among oxidative ezymes ascorbate peroxidase and enzymes that contain heme are acknowledged to convert H_2_O_2_ into molecular oxygen and water by using ascorbic acid (Akram et al., 2017).

#### Phenols

Among different plant metabolites, plant phenolics are of primary importance as they are associated with resistance against parasites, pathogens and predators as well as ultraviolet radiation. They are the basic component of human diet as they are ubiquitous in plant organs (Michalak, 2006). Phenols and polyphenols consist of secondary metabolites with dissimilar functions, for example flavonoids, which are rich in leaves and reproductive organs of the plant, primarily in vacuoles and apoplast. Because of their phenolic OH groups, it is more effective *in vitro* than ascorbic acid and tocopherol (Sakihama et al., 2002). Hence, it is not astonishing that plants with a high content of flavonoids have a higher antioxidant capability (Bolda et al., 2011). They can be proton donors or electron donors. They can therefore not only participate in the quenching process of hydrogen peroxide, but also as metal chelators (Gill and Tuteja, 2010). Due to scavenging activity of the phenols they are oxidized to phenoxy radical. This redevelopment could be by enzymatic or non-enzymatic reaction (Sakihama et al., 2002). Abiotic stressors would cause increased synthesis of flavonoids in plants, supporting the defensive effects of phenolic compounds (Michalak, 2006; Table 1).

**TABLE 1:** Stress induced gene regulation and expression of secondary metabolites.

Stress (Biotic or Abiotic)		Gene expression/Metabolic response		
Stress Induce gene regulation/Transcription	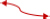	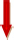		
	
	Secondary metabolites/Signal molecules			
	
	Terpenes	Phenolics		Other metabolites
	Oleanolic acid	Coumarins	Tannins	Quinic acid
	—	Lignin	Caftaric acid	Kynurenic acid
	—	Astringin	Quercetin	Salicylic acid
	—	Kaempferol	Genistein	Methyl salicylate
	—	Hydroxytyrosol glucoside	Hesperidin	Jasmonic acid
	—	—	—	Ethylene

## Role of Plant Enzymes in Maintaining Stress Tolerance

### Superoxide Dismutase

Among enzymatic oxidants super oxide dismutase (SOD) is the first action of defense against ROS enhanced by biotic stress and its reaction products (Alscher et al., 2002; Gill and Tuteja, 2010). SOD is found in many isoforms and these isoforms are classified into three groups accordingly to their active site metal, i.e., FeSOD, Cu/ZnSOD and MnSOD. These are found in specific cell compartments to be exact MnSOD is in the mitochondrial matrix, cell wall and peroxisomes, FeSOD found in plastids, whereas Cu/Zn SOD is localized in cytosol, plastids, peroxisomes and perhaps extracellular space (Kumari et al., 2015). As a leading constituent of the intracellular defense mechanism of plants, SOD is the main enzymatic element of the cellular defense system and resists the enhanced ROS produced by abiotic stress. SOD is abundant in aerobic organisms and acts as catalyst in the dismutation of superoxide into molecular hydrogen peroxide (H_2_O_2_) and oxygen. Under regular situations, peroxidase enzyme and catalase effectively scavenged the resulting H_2_O_2_ (Badawi et al., 2004). In many studies, it has been observed that greater the SOD activity, greater the probability of ROS removal. The up regulation of SOD involves the fight against the overproduction of ROS due to abiotic or biotic stress and is essential for the survival of plants under stressful conditions.

Noteworthy increases in total SOD activity on leaves and some additional SOD isoforms have been reported in several plant species that are subject to various types of abiotic stress in various crops such as tomato, *Arabidopsis*, mulberry (Harinasut et al., 2003), *Hordeum vulgare* (Guo et al., 2004), mustard (Li, 2007; Mobin and Khan, 2007), citrus, *Vigna mungo*, etc. The surplus of SOD transcripts has been detected in reaction to numerous abiotic and biotic stresses to distinguish oxidative stress, which plays an important role in stress tolerance.

**FIGURE 6:**
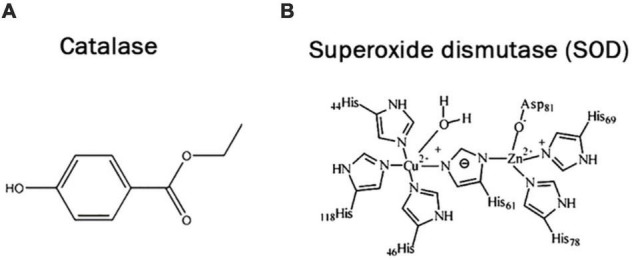
Structural classification of catalase **(A)** and superoxide dismutase **(B)** enzymes.

Transgenic plants that over express various isoforms of SOD increase the improved tolerance to oxidative stress and other ecological stresses. These results have been published in many crops, comprising alfalfa, *Arabidopsis*, potato, rice, tobacco and poplar (Van Camp et al., 1994). Numerous reports have been published on the progress of stress tolerant plants with improved expression of different SODs, i.e., over-expressed Mn-SOD in transgenic *Arabidopsis* (Wang et al., 2004) and tomato (Wang et al., 2007) shown superior salt tolerance, and Cu/Zn-SOD in tobacco expression has shown tolerance to multiple stresses (Figure 6).

### Catalase

Catalase is an enzyme that contains tetrameric heme, present in peroxisomes and related organelles, in which H_2_O_2_ converts to H_2_O and O_2_, which play an important role in antioxidant defense (Garg and Manchanda, 2009). It is an effective ROS sensor and due to its highest reaction rate it can detoxify ROS generated by environmental stresses (Gill and Tuteja, 2010). It’s found in all of the primary H_2_O_2_ producing sites in higher plant’s cells (such peroxisomes, mitochondria, cytosol, and chloroplast). Catalase isozymes come in a variety of molecular forms, indicating its diverse involvement in the plant system. The regulation of H_2_O_2_ by catalase isozymes inside certain cells or organelles at various times and developmental phases interferes with signal transmission in plants, either directly or indirectly. The expression of the cat gene is affected by time, species, and stress. Increased CAT activity appears to be connected to the control of gene expression (Boguszewska and Zagdańska, 2012; Anjum et al., 2022).

## Aptitude of Phytohormones in Stress Tolerance

Phytohormones are low-concentration regulatory chemicals that act as chemical messengers to govern a variety of physiological and developmental processes in plants. They also control internal and external stimuli and play important roles in signal transduction pathways during the stress response (Raza et al., 2019; Sarkar and Sadhukhan, 2022). Auxins (IAAs), cytokinins (CKs), abscisic acid (ABA), gibberellins (GAs), and ethylene are the five major categories of phytohormones (ET). New families of phytohormones include salicylates (SAs), jasmonates (JAs), brassinosteroids (BRs), strigolactones (SLs), polyamines, and certain peptides (Pons, 2020).

Hormones operate as a signaling network in plants, regulating a variety of processes. Crosstalk is the term for the interactions between hormone signal transduction cascades (Mishra et al., 2022; Sarkar and Sadhukhan, 2022). Phytohormones interact via causing a phosphorylation cascade or a shared second messenger to be activated. Furthermore, various phytohormones, including as JA, SA, and ABA, interact to establish a defense network against environmental challenges (Kumudini et al., 2018). Understanding the interactions between phytohormones and defense signaling pathways might lead to the discovery of novel targets for establishing host resistance mechanisms (Kumudini et al., 2018; Nunes da Silva et al., 2022; Table 2).

**TABLE 2:** Role of phytohormones in normal physiological functioning, metabolism and to help plant overcome stresses.

Plant hormones	Formula	Germination	Growth to maturity	Flowering	Fruit development	Abscission	Seed dormancy
Gibberellin	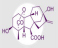	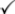	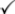	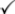	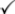	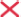	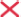
Auxins	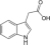	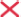	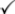	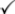	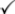	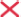	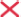
Cytokinins	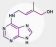	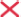	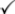	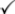	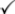	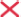	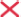
Ethylene	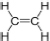	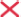	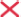	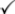	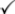	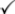	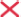
Abscisic acid	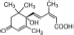	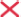	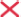	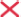	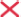	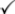	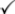

### Abscisic Acid

Abscisic acid (ABA), also known as stress hormone, is involved in the abscission of plant leaves as well as the tolerance of abiotic stimuli (Wani et al., 2016; Kuromori et al., 2022). Seed dormancy, embryo morphogenesis, stomatal opening, cell turgor maintenance, and lipid and storage protein production are only a few of the plant developmental and physiological processes where ABA plays a key role (Anik et al., 2012; Wani et al., 2016; Kumudini and Patil, 2019). ABA affects the expression of genes that code for proteins. Plants can survive in harsh environments (Guimaraes and Jueneman, 2008; Negin and Moshelion, 2016) and water shortages (Atkinson and Urwin, 2012) because of ABA. Under nitrogen shortage (Radin and Mauney, 1986; Délano-Frier et al., 2011; Li S. et al., 2020) and drought stress (Soma et al., 2021), ABA is also crucial for root development and architectural alterations. Dehydrins, osmoprotectants, and protective proteins are all biosynthesized with ABA (Fernando and Schroeder, 2016; Hossain et al., 2022).

### Auxins

One tryptophan-independent and four tryptophan-dependent routes for auxin (IAA) production in plants have been discovered so far (Im Kim et al., 2013; Gruet et al., 2022). IAA is involved in plant growth and development, as well as controlling growth in response to stress (Casanova-Sáez et al., 2021; Kou et al., 2022; Mishra et al., 2022). IAA is important for plant tolerance to salt (Quamruzzaman et al., 2021) and heavy metal stress (Mathur et al., 2022). Auxins also cause the transcription of the major auxin response genes, which have been discovered in rice, Arabidopsis, and soybean (Ahmad et al., 2010). Auxin also controls the interaction of biotic and abiotic stressors (Mathur et al., 2022).

### Salicylic Acid

Salicylic acid (SA), a phenolic compound, regulates the expression of pathogenesis-related proteins (Feng et al., 2020). SA affects plant growth and development, as well as biotic and abiotic stress responses (Hasanuzzaman et al., 2017a,b). SA biosynthesis involves both the major isochorismate (IC) and phenylalanine ammonialyase (PAL) pathways. Low levels of SA increase plant antioxidant capacity (Lefevere et al., 2020). On the other hand, high SA levels have the ability to destroy cells (Verbruggen and Hermans, 2008). The SA contains chaperones, antioxidants, heat shock proteins, and genes involved in secondary metabolite production, such as cinnamyl alcohol dehydrogenase, sinapyl alcohol dehydrogenase, and cytochrome P450 (Verbruggen and Hermans, 2008; Ahmad et al., 2019). Drought responses may be controlled by combining SA and ABA (Gupta et al., 2017; Guo et al., 2021). The SA mechanism in abiotic stress tolerance, on the other hand, is mostly unexplored and requires further study.

### Cytokinins

Plant growth and development is regulated by cytokinins (CKs) (Li et al., 2021). Abiotic stressors, such as salinity and drought, are also a part of their repertoire. They’re also crucial for crop attributes like yield and stress tolerance (He et al., 2018). CKs are likewise abscisic acid antagonists and help seeds come out of dormancy. Reduced CK enhances apical dominance, which aids in drought stress tolerance (Wani et al., 2016).

### Jasmonates

Jasmonates (JAs) are multifunctional phytohormones that arise from the metabolism of membrane fatty acids and are found in a variety of plant species (Singh et al., 2021). Fruiting, blooming, senescence, and secondary metabolism are all processes in which JAs play an important role (Arnao and Hernández-Ruiz, 2020). Salinity, drought, irradiation, and low temperature are all biotic and abiotic stress responses that JAs are engaged in Wang et al. (2020). Exogenous methyl jasmonate (MeJA) concentrations reduce salinity stress symptoms (Taheri et al., 2020). Under salt stress, endogenous levels of JA are also elevated in roots (Ryu and Cho, 2015). Heavy metal stress is also reduced by JA levels because it activates the antioxidant machinery (Jan et al., 2019). MeJA accumulates phytochelatins, which confers resistance to Cu and Cd toxicity (Pál et al., 2018; Romero-Puertas et al., 2019).

### Ethylene

Ethylene (ET) is a gaseous phytohormone that regulates plant growth and development, such as flower senescence, fruit ripening, and petal and leaf abscission, as well as stress responses (Kolbert et al., 2019; Kućko et al., 2022). Sadenosyllmethionine and the cyclic amino acid ACC are used to start ethylene production from methionine. Sadenosyllmethionine is converted to ACC by ACC synthase, whereas ACC oxidase catalyzes the conversion of ACC to ET by ACC oxidase. Endogenous ethylene levels in plants are affected by a variety of abiotic stressors. Stress tolerance is improved when ET levels are higher (Jha et al., 2021; Li et al., 2022). Ethylene, in combination with other hormones such as jasmonates and salicylic acid, regulates plant defense against biotic stress factors (Kumari and Singh, 2022). Plant growth and development may be regulated by a combination of ethylene and abscisic acid (Schaller, 2012; Iqbal et al., 2022).

### Gibberellins

Gibberellins (GAs) are carboxylic acids that have the potential to control plant growth and development (Fàbregas and Fernie, 2022). Leaf expansion, seed germination, stem elongation, flower development, and trichome initiation are all favorably regulated by them (Tamhane et al., 2012). They’re also significant in abiotic stress tolerance, such as osmotic stress (Mujtaba et al., 2021). GAs have the potential to interact with other hormones and influence a variety of developmental processes (Xie et al., 2019; Fenn and Giovannoni, 2021). Both negative and positive regulatory roles may be involved in these interactions (Castorina and Consonni, 2020; Li J. et al., 2020).

### Brassinosteroids

Brassinosteroids (BRs) are polyhydroxy steroidal phytohormones that govern root and stem growth, as well as flower initiation and development in plants (Basit et al., 2021; Manghwar et al., 2022). *Brassica napus* was the source of the first BRs. The most bioactive BRs often employed in physiological research are brassinolide, 24-epibrassinolide, and 28-homobrassinolide (Brosa, 2020). Pollen, fruits, vascular cambium, seeds, leaves, roots, and shoots all contain these (Ali, 2019). Chilling, high temperatures, soil salinity, drought, light, floods, and organic contaminants are all examples of abiotic stress responses that BRs play a part in Wani et al. (2016), Bhuyan et al. (2020), Hossain et al. (2022).

### Strigolactones

Strigolactones (SLs) are carotenoid-derived chemicals that are generated in tiny amounts in roots or manufactured by a variety of plant species (Bhoi et al., 2021). Root architecture and development are influenced by SLs (Marquez-Garcia et al., 2014; Jiu et al., 2022). They can be employed to stimulate parasitic plant seed germination by promoting nodulation during interaction activities (Kapulnik and Koltai, 2014). In addition, SLs have a role in biotic and abiotic reactions (Aliche et al., 2020; Rehman et al., 2021; Brewer, 2022).

## Crosstalk Between Different Metabolic Pathways of Plant Defense System

### Battle of Metabolites of Lipoxygenase Pathway Hormones Against Stress

In the lipoxygenase pathway lipoxygenase was described as an enzyme that oxidizes fatty acids in 1932, but the molecular and functional characterization of these enzymes began in the mid-1990s when jasmonic acid (JA) was one of the basic products of the LOX pathway, has been discovered and studied in the regulation of plant physical and biochemical processes related to stress tolerance (Bajguz and Hayat, 2009; Suardiaz et al., 2016). Lipoxygenase family of the plants catalyzes the regio- and stereospecific adding of molecular oxygen into 1,4-cis, cis-pentadiene remains of linoleic acid, linolenic acid and alpha-linolenic acid (Heshof, 2015). The hydroperoxide product obtained by this process contains a conjugated cis-trans complex which is produced by migration of a double bond during the catalytic cycle. The oxidation of linoleic acid is the first part of the branched enzyme cascade, which results in the production of biologically active compounds, oxylipins (Schaich, 2013). They are involved in the response of plant organisms to abiotic and biotic stress effects, the regulation of senescence and aging and apoptosis. The substrate of lipoxygenase is a free polyunsaturated fatty acid whose content increases after stress (Babenko et al., 2017). Among the components involved in the formation of adaptive responses, compounds in the Lipoxygenase pathway (LOX pathway) oxidized by polyunsaturated fatty acids (PUFAs) play an important role in the main features of the signaling system: perception, conversion and improvement of the signal and expression (La Camera et al., 2004). Metabolites of the LOX pathway of defense genes, i.e., oxylipins interact with other signaling pathways in plant cells, including the signaling pathways of Phytohormones, gibberellin, ethylene, abscisic acid (ABA) and salicylic acid (SA) (Zhang et al., 2016).

**FIGURE 7:**
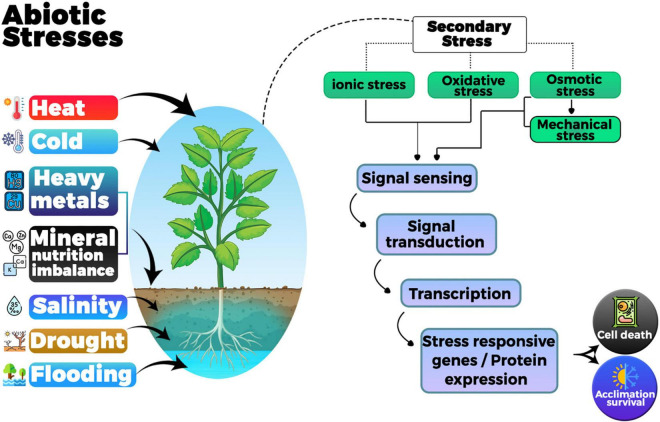
How defense mechanism activates in plants in response to abiotic and biotic stress.

Many studies have revealed that LOX activity upsurges with mechanical injury, ozone effects, hyperthermia, inducers, or elicitors, etc. LOX metabolism is enhanced by the activation of transcription of genes encoding various enzymes. There is adequate information to undertake that the lipoxygenase pathway of transformation of membranous lipids is a pathway which has independent signaling (Rouzer and Marnett, 2011). Like other signaling systems, the major interaction between signaling signals and plasma membrane receptors activates membrane-bound proteins that provide signaling along the signaling pathway. The signal of the lipoxygenase circuit is enhanced by an autocatalytic cycle including calmodulin ions and calcium (Parekh, 2008). The hydroperoxides produced in the plasma membrane (Plasmolemma) from linoleate and linolenat transport calcium ions (Ca^2+^) from the outside to the inside of the cell (Blokhina et al., 2003). Rise in the concentration of cytosolic calcium ions results in the activation of phospholipase A and the release of polyamine fatty acids (PUFA) from the phospholipid (Kacperska, 2004). In general, LOX, a key enzyme in the cascade, regulates LOX gene expression through cascade end products. Intermediate and final LOX metabolism products can activate protein kinases, send signals, and transduce them (Barenstrauch, 2018). Oxylipins induces the formation of proteins and enzymes as well as plant antibiotics involved in detoxification in the presence of pathogens. The oxylipins family contains jasmonic acid and its direct precursor, 12-oxo-plant dienoic acid (OPDA), formed enzymatically and subjected to various stresses, including wounds and pathogens (Grun et al., 2007). Although the molecular mechanisms of oxylipins gene activation have not been well studied, available data indicate that these compounds can express protein genes involved in the formation of stress tolerance in plants (Barenstrauch, 2018; Figure 7).

These both prompt the synthesis of protease inhibitors and participate in the release and accumulation of alkaloids, providing selective inhibition of polypeptide synthesis. The synthetic jasmonic acid (JA), which produces the formation of reactive oxygen species and stimulates certain protective compounds associated with pathogenesis, induces resistance to plant diseases. JA has been shown to be involved in signaling the surface of infected cells to the nucleus and between cells, which has promoted the expression of protective genes. Wherever it’s the situation of stress Jasmonic acid reaction or response is controlled by JASMONATE-ZIM-DOMAIN (JAZ) repressor, which is a set of protein. These repressors act together with F box protein CI1 which is a fundamental part of skp-Cullin-F box complex involved in co-reception of biologically active Jasmonic acid (JA-Ile) (Yang et al., 2007; Chung et al., 2008). When plants are mechanically damaged, abscisic acid (ABA) also appears to have a positive effect on LOX activity. Exogenous ABA has been shown to stimulate lipoxygenase activity, activate peroxide oxidation of membrane lipids, and contribute to tolerance when rice leaves are injured. A positive correlation between ABA concentration and LOX transcript content is revealed in water-stressed plants. Mechanical injuries cause LOX activity and ABA and JA levels increase (Hassanein et al., 2009). Generally, salicylic acid is responsible for local and systemic defense response for biotrophic pathogens as well as systemic acquired resistance (SAR), While ethylene and jasmonic acid mediate responses to necrotrophs (Durrant and Dong, 2004; Glazebrook, 2005). Salicylic acid, jasmonic acid and ethylene also crosstalk antagonistically or synergistically with eachother among pathways (Spoel et al., 2007). The initial actions of plant adaptation to environmental stress are stress signal detection and consequent signal transduction by ABA-dependent or ABA-independent pathways leading to the activation of several physiological and metabolic responses. ABA phytohormone is a central regulator of abiotic stress, particularly plant resistance to drought, and coordinates complex genetic regulatory networks to allow plants to cope with reduced water use (Cutler et al., 2010). ABA-dependent signaling systems have been described as pathways involved in stress adaptation by inducing at least two distinct regulons (a set of genes controlled by a definite TF). One of which is AREB/ABF (ABA responsive element binding protein/ABA binding factor) and second one is MYC (myeloid cell oncogene)/MYB (oncogenic myeloblastosis) regulon (Shinozaki and Yamaguchi-Shinozaki, 2000). Salicylic acid is an early signaling molecule in the temperature-stress response. This is important for a variety of reasons. Salicylic acid plays a fundamental role in the systemic acquired resistance to pathogens such as bacteria, fungi, and viruses (Schmelz et al., 2003), and the increase in salicylic acid levels is positively correlated with the level of resistance to pathogens in plants. It has been demonstrated that exogenous application of salicylic acid or acetylsalicylate improves tolerance against heat (Heil and Bostock, 2002).

In addition, many biologically active oxylipins are formed non-enzymatically by the action of reactive oxygen species (ROS), which also accumulate in response to infection by a pathogen, heavy metals, and other stress (Farmer and Davoine, 2007). It is increasingly evident that non-enzymatically formed oxylipins, including hydroxylated fatty acids and phytoprostans, have also been shown to activate the expression of stress response genes, thus enhancing protection against subsequent oxidative stress (Sattler et al., 2006). Physiologically active oxylipins which are called traumatic acid and traumatin are also capable of inducing division of cells and callus formation on impaired or damaged sites (Sattler et al., 2014). Another stress tolerant hormone is brassinosteroids which are plant specific phytohormones and they are very significant because of their multipurpose roles in plants. Many studies have been reported on the capability of brassinosteroids for improving stress tolerance (Wani et al., 2016). But their principal mechanisms of action are still mysterious. This may be due to the integration of brassinosteroid indications into many other signal networks related to stress moderation (Divi et al., 2016).

### Role of PR Proteins in Attaining Stress Tolerance

In reaction to attacking pathogens or stress, the creation and accretion of pathogenic proteins in plants is so essential. Phytoalexins are primarily manufactured by healthy cells nearby to locally injured and necrotic cells, but pathogen related proteins (PR proteins) mount up locally in damaged and surrounding tissues as well as in remote non-infected tissues (Heil and Bostock, 2002). In uninfected parts of the plant the production of PR proteins of the plant prevents additional infection of the affected plants. The PR protein in plants has been discovered and reported for the first time in plants of tobacco which are infected with tobacco mosaic virus. These PR proteins were found in many other plants after that (Yalpani et al., 1991). Most of the PR proteins found in plants are soluble in acid, have low molecular weight and are protease resistant. Based on isoelectric point PR proteins could be acidic or basic proteins, but they are similar in function (Ebrahim et al., 2011). Presently, PR proteins are classified into 17 families according to their nature and function, comprising β-1,3-glucanase, chitinase, thaumain-like protein, peroxidases and ribosome interacting proteins, defenses, thionins, non-specific lipid transfer proteins, oxalate oxidase and oxalate oxidase proteins. Among these PR proteins, chitinase and β-1,3-glucanase are two important hydrolases, which are found abundantly in numerous plant species after being infected with different kinds of pathogens (Van Loon et al., 2006).

Several pathogens pass this first defense obstacle, and they must have another means of defense against these pathogens. Likewise, defense mechanism is constituted by pathogen-induced defense reactions, which include allergic reactions followed by reactive oxygen species, cross-linking of cell wall, production of antibiotic molecules such as phytoalexins and ultimately the construction of PR proteins (Van Baarlen et al., 2007). Among them, the PR protein, which is a key component of SAR, which is a inducible immune response of the plant that prevents further infection of the uninfected portion of the host. The term “PR protein” refers to a collection of different proteins induced by phytopathogenic agents as well as signaling molecules related to defense. Activation of the defense signaling pathway occurs after pathogen challenge, namely salicylic acid and jasmonic acid, which leads to PR protein accumulation (Xu et al., 1994). This minimizes pathogen capacity or disease attack in uninfected plant organs. There are two kinds of pathogens, biological and dead nutrients, the first to activate the salicylic acid pathway, which excites the transcription of NPR1 (a non-gene 1 expression associated with the pathogen), which leads to activation and accumulation. The salicylic acid signature genes (PR1, PR2, and PR5) locally and systemically generate systemic acquired resistance (SAR). The second dead tropical pathogen stimulates the jasmonic acid pathway to induce activation of jasmonic acid signature genes (PR3, PR4, and PR12) and causes to local accretion of its products, thus providing only locally acquired resistance (LAR) (Gruner et al., 2013).

SAR offers increased resistance to variety of pathogens (Fu and Dong, 2013). In addition, PR proteins are broadly disseminated in plant domains and are found in all organs of the plant that are especially rich in leaves and form 5 to 10% of the total leaf protein (Van Loon et al., 1994). These proteins have been effectively separated from different types of the plant affiliated to various families (Takeda, 1991). Based on the biochemical characteristics, PR proteins vary greatly from one another. They are normally low molecular weight proteins of around 6 to 43 kDa, are thermostable, resistant to protease and stay soluble at low pH (Van Loon et al., 1994).

PR proteins categorized into two subgroups, an acid PR protein that is normally secreted in the extracellular space and a second subgroup is a simple PR protein that is usually transported in the vacuole by means of a signal sequence at the C-terminal end (Takeda, 1991). Proteins associated with pathogenesis store mainly in the apoplastic domain, but they are also vacuolar (Van Loon et al., 1994). Transcriptomic studies showed that the PR gene is strongly provoked by abiotic and biotic stresses, making it one of the best nominees for the development of various varieties of stress tolerant crops (Sreenivasulu et al., 2007; Atkinson and Urwin, 2012). There are two means of plant immunity: immunity triggered by a pathogen-associated molecular pattern and effector-triggered immunity (ETI). Pathogen-associated molecular models (PAMPs) typically consist of microbial or pathogenic structures, such as flagellin, lipopolysaccharide, and fungal cell wall constituents (chitin and dextran), referred to as plant recognition receptors (PRRs). Body recognition also activates PTI (pattern triggered immunity) (Zipfel and Felix, 2005). Contrarily, microbial pathogens secrete effector proteins, recognized by a specific set of resistance proteins (R), that arouse the activation of induced defense responses, named ETIs (Effector triggered immunity) (Dangl and Jones, 2001).

These effector proteins are main components of the virulence of fungal pathogens for plants and are mostly important in the biological nutrition stage of infection (Sonah et al., 2016). Though, the importance of PR proteins in plant-pathogen communications has been extensively recognized and an increasing number of established pathogenic effector proteins intermingle directly with PR proteins during infection (Breen et al., 2017). The complication and effectiveness of plant defense systems against pathogen attacks differ among plant species (Jones and Dangl, 2006).

**FIGURE 8:**
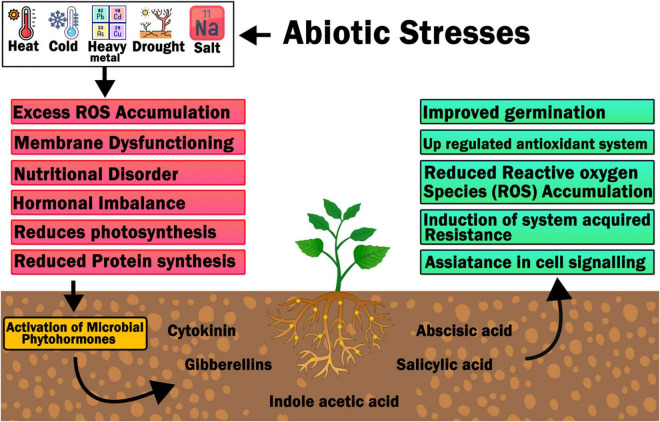
Effect of abiotic stress on plant’s normal physiological functions, activation of phytohormones and response of plant to stress: Story of stress tolerance in plants.

There are some PR proteins which are alleged antimicrobial peptides. Those are cysteine rich molecules that have antimicrobial activity. AMP is abundant in nature and is a significant element of host defense contrary to various and pests and microbial pathogens, from microorganisms to dissimilar biological forms of plants (Lemaitre and Hoffmann, 2007; Figure 8).

### An Overview of Some Vital Metabolites Associated With Plant Stress Tolerance

Based on existing information and knowledge of signaling pathways of stress, any universal trail for abiotic stress can be separated into the succeeding main stages: such as signal perception leading to signal transduction then expression of stress response genes, and activation physiological and metabolic response. During this procedure, firstly plant cells perceive stress stimuli through receptors or sensors present in the cell wall or cell membrane.

The apprehended extracellular signs are then transformed into intracellular signals by a second messenger, comprising calcium ions (Ca^+2^), inositol triphosphate (IP3), reactive oxygen species (ROS), cyclic nucleotides, i.e., cAMP and GMP, sugars and nitric oxide.

The corresponding signaling pathway initiate by second messenger to transduce the signal (Bhargava and Sawant, 2013). Phosphorylation is mediated by protein kinases and dephosphorylation is mediated by phosphatases in most of the pathways of signal transduction. Many different kinds of metabolites such as all compatible solutes especially glycine betaine and proline, antioxidants both enzymatic and non-enzymatic (for example glutathione, tocopherol, ascorbic acid, phenol, superoxide dismutase), polyamines (putrescine, spermidine, spermine), lipoxygenases including oxylipins, jasmonic acid, abscisic acid, methyl jasmonate, salicylic acid, ethylene, brassinosteroids, traumatin get activated in response to enhanced production of ROS work together by cross linking each other to overcome stress caused by biotic and abiotic factors. PR proteins come in action in case of pathogenic attack ad these are stimulated by hormones such as jasmonic acid and salicylic acid.

This eventually activates SAR (systemic acquired resistance) and results in provoking immunity in plants. Ashraf and Foolad (2007), Takahashi and Kakehi (2010), Peleg and Blumwald (2011), Huang et al. (2012), Puranik et al. (2012) and a cascade of regulatory mechanism starts; resulting which transcription factors (TF) get triggered. In addition, they muddle specifically to cis elements in the promoter of stress-sensitive genes and regulate their transcription (Dhanush et al., 2015). At the same time, the transcription factors themselves are up regulated by other upstream components at the transcriptional level and various post-transcriptional modifications like ubiquitination and sumolylation form a complex network of regulators to regulate the expression of the stress response genes which in turn determines stimulation of physiological and metabolic reactions (Mizoi and Yamaguchi-Shinozaki, 2013).

Phytoalexins and PR proteins are produced specially in case of biotic stress such as pathogen attack which leads to SAR (systemic acquired response) pathway and ultimately activation of transcription factors and thus stress tolerance occurs by expression of stress response genes. All the above components, from the most important downstream functional gene receptors, are a versatile pathway for stress signaling in plants (Table 3).

**TABLE 3:** Enhancement of secondary metabolites under stress to cope with stressed conditions in plants.

Stress	Production of secondary metabolites	Response of plants	References
Drought	Phenolics	Activates antioxidant property of plants, PAL enzyme activity Provide defense against microbial pathogens, insect pests and large herbivores	García-Calderón et al., 2015; Rezayian et al., 2018;
	Anthocyanins/Flavonoids/Amino acids/Alkaloids	Increase antioxidant activity and anti-inflammatory response, affects transamination, glycolysis, citric acid cycle and glutamate- mediated proline biosynthesis and promotes ripening of leaves	Cakir and Cebi, 2010; Bowne et al., 2012; Guo et al., 2018;
	Terpenes	Increase in photosynthesis and leaf water status, alleviate oxidative damage on cellular membrane, Increase defense against phytophagous insects, fungi and microbes	Turtola et al., 2003
Heat Stress	Polyamines phenyl amides	Increases defense	Edreva et al., 2008
Cold stress	Phenolics	Increases defense	Griffith and Yaish, 2004
Antifungal	Phenolics	Increases defense	Mishra et al., 2006; Jain et al., 2015
Antiviral	Phenolics	Increases defense	Özçelik et al., 2011
Salt stress	Polyphenolic alkaloids	Increases defense	Martínez-Ballesta et al., 2014; Rivero et al., 2018
Light stress	Triterpenes	Increases defense	Fournier et al., 2003
Anti-insect	Monoterpenes	Increases defense	Ahmed et al., 2017
Anti-herbivores	Amino acids, Glucoside	Increases defense	Moulin et al., 2006; Mazid et al., 2011

## Molecular Prospects of Role of Metabolites in Plant Stress Tolerance

Adverse conditions in the natural world are always a result of a mix of stressors which include water limitation, high temperature or irradiation and high osmolality etc. This is why determining which stress component is responsible for eliciting a certain physiological response is usually challenging. Researchers have historically subjected plants to a single stress factor under carefully controlled settings while keeping the rest of the parameters at optimal values, therefore, ignoring their impact to physiological responses (Chaves et al., 2009). Plants physiological responses to environmental signals include changes not just in transcription but also in post-translational protein modifications and metabolite alteration and/or accumulation, all of which contribute to a specific physiological response or phenotype (Verslues et al., 2006; Sami et al., 2018). Stress physiological responses in plants are geared toward stress tolerance, sensitivity, or avoidance (Cattivelli et al., 2008; Baldoni, 2022). A unique gene expression profile determines the actual metabolite composition of certain plant species. Precursors and intermediates are funneled into a bioactive molecule when a metabolic pathway is triggered, such as an antioxidant, a signaling agent, a cell structure biosynthesis step, or even a storage component. Other chemicals (signaling molecules like plant hormones) or intermediates that can feedback activate or inactivate other metabolic pathways can control the synthesis of these compounds (Kerchev et al., 2012; Wishart, 2019).

It is difficult to find molecular features that differ in response to stressors. Metabolic profiling might be used to describe molecular attributes involved in stress response, which would be useful for breeding efforts (Bueno and Lopes, 2020). Certainly, metabolomics is a useful tool for finding biological or physiological reactions to environmental changes, especially when used in conjunction with other ‘omics’ methods like transcriptomics and proteomics (Amiour et al., 2012; Carrera et al., 2021). In plants under abiotic stress, metabolomics is utilized to detect and/or quantify main and secondary stress-responsive compounds (Isah, 2019). To study metabolic reprogramming in plants under abiotic stress, two primary techniques, i.e., non-targeted and targeted are utilized (Mishra et al., 2015; Mashabela et al., 2022). Non-targeted metabolomics give a broad picture of the most abundant metabolites in plants under a variety of stress conditions. Under varied environmental conditions, targeted metabolomics identify, assess, and analyze known compounds in plants (Van Meulebroek et al., 2016; Feng et al., 2022).

## Conclusion

Over the past few years, the understanding of the importance of metabolic adjustment in plants under adverse environmental conditions has improved dramatically. Stress tolerance in plants through natural means is a very complicated process which involves networking among multiple metabolites of various metabolic pathways. The analysis of metabolic adjustment and transgenic methods for plants with altered levels of tolerance of the stress provides important additional evidence to improve understanding of the role of unalike metabolites involved in adapting to challenging situations. Major metabolites involved in battling against environmental stresses are glutathione, tocopherol, ascorbic acid, phenolics, superoxide dismutase, glycine betaine, proline, putrescine, spermidine, spermine, oxylipins and its derivatives such as jasmonic acid, methyl jasmonate and other hormones like salicylic acid, brassinosteroids, ethylene, the involvement of traumatin also cannot be denied in this regard. PR proteins also play important role on combating biotic stresses. The review focuses on the regulatory mechanisms of vital metabolites associated with plant resistance under adverse conditions. It also explores the cross-talk between different metabolic pathways which are mainly regulated due to stress-induced changes in plant defense system.

## Future Prospective

Despite these important advances, a number of problems still need to be resolved, such as, the developmental stage of plant and cellular metabolism during development directly associated with plant stress response during unfavorable conditions. Hence, it would be remarkable to examine how different developmental phases of plants affect the metabolic regulation under stress conditions. Non-etheless, it is also necessary to dissect stress response mechanisms by identifying the main regulatory factors associated with stress tolerance in stress-resistant genetically engineered plants.

## Author Contributions

AH, BM, SN, SI, and MK: conceptualization. DC, BM, MS, and GD: methodology. GD and MK: software. AH: validation. BM: formal analysis. AH, AA, BM, SN, SI, and MK: investigation. DC, MS, and GD: resources. MR, MA, AE-S, HE, and EM: data curation. BM, AH, SN, MK, and SI: writing—original draft preparation. DC, MS, GD, MR, MA, AA, AE-S, HE, and EM: review and editing. SN and MK: supervision. AH, BM, SN, and MK: project administration. All authors have read and agreed to the published version of the manuscript, and publishing consent.

## Conflict of Interest

The authors declare that the research was conducted in the absence of any commercial or financial relationships that could be construed as a potential conflict of interest.

## Publisher’s Note

All claims expressed in this article are solely those of the authors and do not necessarily represent those of their affiliated organizations, or those of the publisher, the editors and the reviewers. Any product that may be evaluated in this article, or claim that may be made by its manufacturer, is not guaranteed or endorsed by the publisher.
