# Corrigendum: Role of Promising Secondary Metabolites to Confer Resistance Against Environmental Stresses in Crop Plants: Current Scenario and Future Perspectives

**DOI:** 10.3389/fpls.2022.950612

**Published:** 2022-06-14

**Authors:** Delai Chen, Bismillah Mubeen, Ammarah Hasnain, Muhammad Rizwan, Muhammad Adrees, Syed Atif Hasan Naqvi, Shehzad Iqbal, Muhammad Kamran, Ahmed M. El-Sabrout, Hosam O. Elansary, Eman A. Mahmoud, Abdullah Alaklabi, Manda Sathish, Ghulam Muhae Ud Din

**Affiliations:** ^1^College of Life Science and Technology, Longdong University, Qingyang, China; ^2^Gansu Key Laboratory of Protection and Utilization for Biological Resources and Ecological Restoration, Qingyang, China; ^3^Institute of Molecular Biology and Biotechnology, The University of Lahore, Lahore, Pakistan; ^4^Department of Environmental Sciences and Engineering, Government College University Faisalabad, Faisalabad, Pakistan; ^5^Department of Plant Pathology, Bahauddin Zakariya University, Multan, Pakistan; ^6^Faculty of Agriculture Sciences, Universidad de Talca, Talca, Chile; ^7^School of Agriculture, Food and Wine, The University of Adelaide, Adelaide, SA, Australia; ^8^Department of Applied Entomology and Zoology, Faculty of Agriculture (EL-Shatby), Alexandria University, Alexandria, Egypt; ^9^Plant Production Department, College of Food and Agricultural Sciences, King Saud University, Riyadh, Saudi Arabia; ^10^Department of Food Industries, Faculty of Agriculture, Damietta University, Damietta, Egypt; ^11^Department of Biology, Faculty of Science, University of Bisha, Bisha, Saudi Arabia; ^12^Centro de Investigación de Estudios Avanzados del Maule (CIEAM), Vicerrectoría de Investigación y Postgrado, Universidad Católica del Maule, Talca, Chile; ^13^State Key Laboratory for Biology of Plant Disease and Insect Pests, Institute of Plant Protection, Chinese Academy of Agricultural Sciences (CAAS), Beijing, China

**Keywords:** PR proteins, polyamines, compatible solutes, antioxidants, stresses

In the published article, there is a production house-based error in the affiliation of “Muhammad Rizwan^3^.” Instead of “Muhammad Rizwan^3^,” it should be “Muhammad Rizwan^4^.”

The authors apologize for this error and state that this does not change the scientific conclusions of the article in any way. The original article has been updated.

## Publisher's Note

All claims expressed in this article are solely those of the authors and do not necessarily represent those of their affiliated organizations, or those of the publisher, the editors and the reviewers. Any product that may be evaluated in this article, or claim that may be made by its manufacturer, is not guaranteed or endorsed by the publisher.

